# Supplementary material for: RNA tertiary structure and conformational dynamics revealed by BASH MaP
Source: eLife. 2024 Dec 3;13:RP98540. doi: 10.7554/eLife.98540 (PMC11614387; doi:10.7554/eLife.98540)
Supplement: Supplementary file 1. [file elife-98540-supp1.docx]

**Supplementary Table 1 | Spinach G-quadruplex identification false positive rate estimation for various BASH MaP data processing approaches**

| **BASH MaP N7G reactivity (population average) (choose 9 lowest reactivity G’s)** | | | **BASH MaP N7G reactivity (DANCE state 1) (choose 9 lowest reactivity G’s)** | | |
| --- | --- | --- | --- | --- | --- |
| **Sensitivity** | **PPV** | **FPR** | **Sensitivity** | **PPV** | **FPR** |
| 88.88% (8/9) | 88.88% (8/9) | 11.1% (1/9) | 100% (9/9) | 100% (9/9) | 0% (0/9) |

| **BASH MaP N7G reactivity (tertiary folding constraint method)** | | |
| --- | --- | --- |
| **Sensitivity** | **PPV** | **FPR** |
| 77.77% (7/9) | 100% (7/7) | 0% (0/7) |

**PPV = Positive predictive value**

**FPR = False positive rate**
